# Association between breech presentation in twins and developmental dysplasia of the hip

**DOI:** 10.1007/s00132-026-04867-0

**Published:** 2026-07-14

**Authors:** Beatrice Jung, J. Hollfelder, V. zur Strassen, B. Bodelle, M. Schulz

**Affiliations:** 1 Zentrum für Orthopädie und Unfallchirurgie, Universitätsmedizin Mainz, Mainz, Germany; 2 Institute of Diagnostic and Interventional Radiology, University Hospital Frankfurt, Frankfurt am Main, Germany; 3 Department of Radiology, Klinikum Mutterhaus der Borromäerinnen, Trier, Germany; 4 Department of Emergency Medicine, St. Marienkrankenhaus Ludwigshafen, Ludwigshafen, Germany

**Keywords:** Hip ultrasonography, Graf classification, Neonatal screening, Cesarean section, Fetal presentation, Hüftsonographie, Graf-Klassifikation, Neugeborenenscreening, Kaiserschnitt, Fetale Lage

## Abstract

**Background:**

Breech presentation is considered one of the main risk factors for developmental dysplasia of the hip (DDH) in singleton pregnancies; however, data regarding twins are limited. The aim of this study was to evaluate whether breech presentation in twins is associated with abnormal hip ultrasound findings and to assess the influence of delivery mode, sex and gestational age.

**Methods:**

Obstetric and ultrasound data from twin births at Frankfurt University Hospital between 2010 and 2014 were retrospectively analyzed. A total of 130 twins in whom at least 1 infant with breech presentation and both infants underwent hip ultrasound according to Graf’s technique within the first 14 days after birth were included. Twins were divided into breech-breech, breech-vertex and breech-transverse groups. Hip findings were categorized as normal (Graf type Ia/Ib) or abnormal/immature (Graf type IIa or higher). Statistical analysis was performed using Fisher-Freeman-Halton exact testing.

**Results:**

No infant demonstrated manifest DDH (Graf type IIc or higher). Overall, 5.4% of infants showed physiologically immature or abnormal hip ultrasound findings classified as Graf type IIa. No significant association was found between breech presentation and abnormal hip findings; however, abnormal findings were more common in the breech-transverse group compared to the breech-breech group (*p* = 0.037). Infants delivered via cesarean section demonstrated a higher rate of abnormal findings than vaginally delivered infants (*p* = 0.041).

**Conclusion:**

In this cohort, breech presentation in twins was not associated with an increased rate of abnormal hip ultrasound findings. The combination of breech and transverse presentation may represent a higher risk constellation. Due to the retrospective design and lack of a singleton control group, conclusions regarding a modifying effect of twin pregnancy on the occurrence of DDH cannot be drawn.

**Level of evidence:**

Retrospective cohort study. Level III evidence.

## Abstract (de)

**Hintergrund:**

Die Steißlage gilt als einer der Hauptrisikofaktoren für die Hüftdysplasie („developmental dysplasia of the hip“, DDH) bei Einlingsschwangerschaften; für Zwillinge sind jedoch nur eingeschränkt Daten verfügbar. Ziel der vorliegenden Studie war es zu untersuchen, ob die Steißlage bei Zwillingen mit auffälligen Hüftbefunden in der Sonographie einhergeht, und den Einfluss der Entbindungsart, des Geschlechts und des Schwangerschaftsalters zu ermitteln.

**Methode:**

Geburtshilfliche und sonographische Daten von Zwillingsgeburten am Universitätsklinikum Frankfurt zwischen 2010 und 2014 wurden retrospektiv ausgewertet. In die Studie wurden 130 Zwillinge einbezogen, von denen mindestens einer eine Steißlage aufwies und wo bei beiden innerhalb von 14 Tagen nach der Geburt eine Sonographie der Hüfte gemäß der Technik von Graf durchgeführt worden war. Die Zwillinge wurden eingeteilt in Gruppen mit Steiß-Steiß-Lage, Steiß-Kopf-Lage und Steiß-Quer-Lage. Die Hüftbefunde wurden kategorisiert als normal (Graf-Typ Ia/Ib) oder auffällig/unreif (Graf-Typ IIa oder höher). Eine statistische Analyse wurde unter Verwendung des Fisher-Freeman-Halton-Exact-Tests durchgeführt.

**Ergebnisse:**

Keines der Neugeborenen wies eine manifeste DDH auf (Graf-Typ IIc oder höher). Bei 5,4 % der Neugeborenen wurden aus physiologischer Sicht unreife oder auffällige Hüftbefunde in der Sonographie festgestellt, die als Graf-Typ IIa klassifiziert wurden. Es wurde kein signifikanter Zusammenhang zwischen Steißlage und auffälligen Hüftbefunden beobachtet; jedoch waren auffälligen Befunde häufiger in der Gruppe mit Steiß-Quer-Lage als in der Gruppe mit Steiß-Steiß-Lage (*p* = 0,037). Durch Kaiserschnitt entbundene Neugeborene wiesen eine höhere Rate an auffälligen Befunden auf als vaginal entbundene Neugeborene (*p* = 0,041).

**Schlussfolgerung:**

In der vorliegenden Kohorte gab es keinen Zusammenhang zwischen der Steißlage bei Zwillingen und einer erhöhten Rate an auffälligen Hüftbefunden in der Sonographie. Die Kombination von Steißlage und Querlage stellt möglicherweise eine Konstellation mit einem höheren Risiko dar. Aufgrund des retrospektiven Designs und des Mangels an einer Kontrollgruppe von Einlingen können keine Schlussfolgerungen hinsichtlich eines modifizierenden Effekts der Zwillingsschwangerschaft auf die DDH gezogen werden.

**Evidenzlevel:**

Retrospektive Kohortenstudie. Level-III-Evidenz.

## Introduction

Developmental dysplasia of the hip (DDH) is a congenital disorder of the hip joint that can be detected even in early infancy. One of the most established screening methods for DDH is ultrasonography according to Graf’s technique, which provides a standardized assessment of neonatal hip morphology [1].

In Germany, routine hip ultrasonography is performed during the mandatory U3 examination at the age of 4–5 weeks [2]. At Frankfurt University Hospital (FUH), infants with known risk factors for DDH, such as breech presentation or a positive family history, additionally undergo hip ultrasonography during the U2 examination within the first 14 days after birth. Early diagnosis enables prompt treatment and can reduce long-term complications; however, physiologically immature hips detected shortly after birth can still mature spontaneously during the first weeks of life and therefore require follow-up rather than immediate treatment.

Breech presentation is considered one of the major risk factors for DDH in singleton pregnancies [3–7]. In addition, factors associated with restricted intrauterine fetal mobility, such as oligohydramnios, have also been linked to an increased risk of DDH [4, 8, 9]. As fetal mobility can likewise be reduced in twin pregnancies and noncephalic presentation is common in twins, occurring in approximately 60% of cases [10], twin pregnancy has been discussed as a possible factor influencing the occurrence of DDH; however, previous studies investigating DDH in twins have reported conflicting results, and some authors even described lower rates of DDH in twins compared to singletons [11].

Prenatal sonographic studies suggest that abnormalities of hip development can already be detectable during the third trimester, supporting the concept that DDH predominantly develops prenatally rather than during delivery [12]. Nevertheless, several studies have investigated the association between delivery mode and DDH in breech-presenting singletons. While some authors reported lower rates of DDH after cesarean section compared to vaginal delivery, the underlying mechanisms remain unclear. It has been suggested that delivery mode may influence postnatal hip instability or the detection rate of pre-existing dysplasia rather than the development of DDH itself [13–16].

Therefore, the aim of this study was to evaluate the association between breech presentation in twins and abnormal hip ultrasound findings and to investigate the possible influence of delivery mode, sex, and gestational age on neonatal hip morphology.

## Material and methods

For this retrospective cohort study, data from all twin births at Frankfurt University Hospital (FUH) between 2010 and 2014 were reviewed. Twins in whom at least one infant presented in breech position at birth were considered eligible for inclusion, resulting in an initial cohort of 432 infants.

**Fig. 1:**
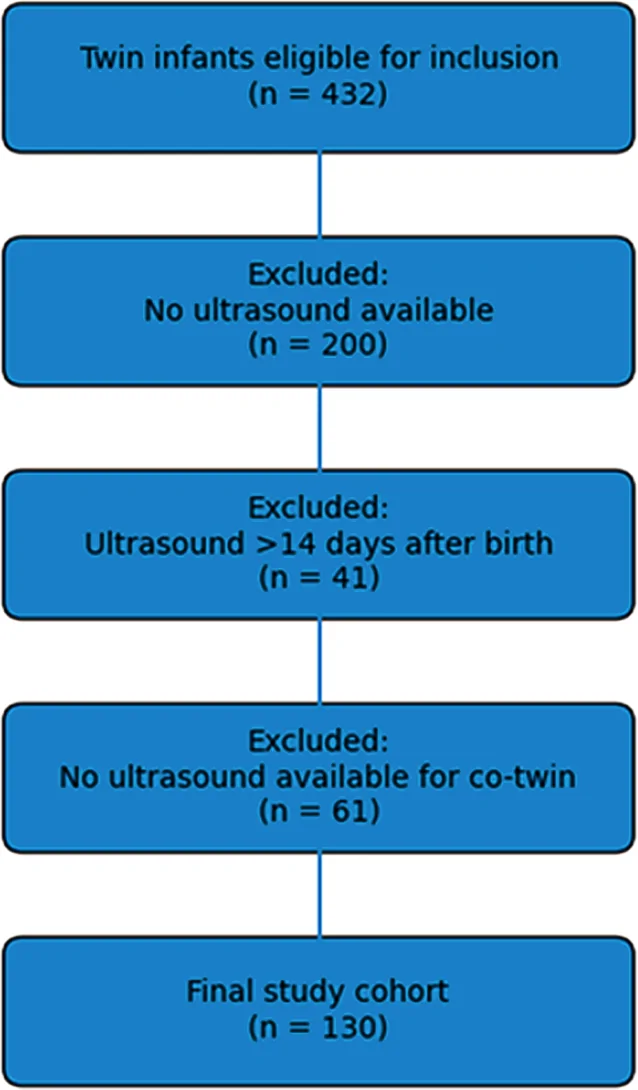
Flowchart illustrating patient selection and exclusion criteria for the final study cohort

Only twins who underwent hip ultrasonography according to Graf’s technique within the first 14 days after birth were included in the final analysis. As hip ultrasonography during the U2 examination was primarily performed in infants considered at increased risk for DDH, not all twins born during the study period underwent early ultrasound examination. Consequently, 200 infants without available ultrasound examinations had to be excluded. In addition, 41 infants were excluded because ultrasound examination had been performed more than 14 days after birth and therefore did not meet U2 examination criteria. Furthermore, 61 infants were excluded because no corresponding ultrasound examination was available for the co-twin. The final study population consisted of 130 infants. The inclusion and exclusion process is summarized in Fig. 1.

In addition to hip ultrasound findings, obstetric data including presentation at birth, delivery mode, sex, and gestational age were obtained from the medical records. Information regarding family history of DDH and detailed subtypes of breech presentation (e.g., frank breech versus complete breech) was not consistently available and therefore could not be included in the analysis.

To investigate the association between fetal presentation and abnormal hip findings, the three presentation types, i.e., vertex, breech and transverse, were compared. Hip findings were categorized as normal (Graf types Ia and Ib) or abnormal/immature (Graf type IIa or higher). In cases of differing findings between the left and right hip, the more severe classification was used for analysis.

The infants were additionally divided into three groups according to the presentation combination within each twin pair: breech-breech, breech-vertex, breech-transverse. Subsequently, hip findings were compared between these groups. As female sex has previously been identified as a risk factor for DDH, hip findings were also analyzed according to sex [3–6]. Furthermore, delivery mode was analyzed by comparing vaginal delivery and cesarean section. Instrumental vaginal deliveries were included in the vaginal delivery group. As preterm birth is common in twin pregnancies and can influence the occurrence of DDH, preterm and term infants were additionally compared [17, 18]. Finally, hip findings of all breech-presenting infants were analyzed according to both presentation group and delivery mode. Within each group, findings were compared according to fetal presentation and delivery mode. In the breech-transverse group, all infants had been delivered via cesarean section. Statistical analysis was performed using the Fisher-Freeman-Halton exact test. *P*-values < 0.05 were considered statistically significant. All ultrasound examinations were performed by trained physicians of the Department of Radiology at FUH.

## Results

The distribution of hip types according to Graf classification is shown in Table 1. No infant demonstrated manifest DDH (Graf type IIc or higher). Overall, 5.4% of infants showed physiologically immature or abnormal hip ultrasound findings classified as Graf type IIa. The distribution of normal and abnormal findings according to presentation, twin group, sex, delivery mode, and gestational age is summarized in Table 1.

**Table 1:** Distribution of hip ultrasound findings according to presentation group

Presentation group	Normal findings *n* (%)	Abnormal findings (Graf IIa)* n* (%)	*p*‑value
Breech–breech	100	0	–
Breech–vertex	94.9	5.1	–
Breech–transverse	83.3	16.7	0.037
Overall cohort	94.6	5.4	–

Abnormal findings were defined as Graf type IIa or higher. No infant demonstrated manifest DDH (Graf type IIc or higher).

No statistically significant difference was observed between the three presentation types. Abnormal hip ultrasound findings were present in 5.1% of vertex-presenting infants, 4.9% of breech-presenting infants, and 11.1% of transverse-presenting infants (*p* = 0.528); however, comparison of the three twin groups demonstrated a significant difference between the breech-breech group, in which no abnormal findings were observed and the breech-transverse group, in which abnormal findings were present in 16.7% of infants (*p* = 0.037). No significant difference was found between the breech-breech and breech-vertex groups.

Within the breech-transverse group, no significant difference was observed between breech-presenting infants and their transverse-presenting co-twins (*p* > 0.999).

Normal hip findings were present in 93.0% of female and 96.6% of male infants. This difference was not statistically significant (*p* = 0.455).

Infants delivered via cesarean section demonstrated a significantly higher rate of abnormal hip ultrasound findings compared to vaginally delivered infants (9.0% versus 0%, *p* = 0.041).

No significant association was found between gestational age and abnormal hip findings when comparing preterm and term infants (*p* = 0.709; Table 2).

**Table 2:** Association of clinical variables with abnormal hip ultrasound findings

Variable	Normal findings *n* (%)	Abnormal findings *n* (%)	*p*‑value
Female sex	93.0	7.0%	0.455
Male sex	96.6	3.4	–
Cesarean section	91.0	9.0	0.041
Vaginal delivery	100	0	–

Within the breech-vertex group, no significant difference was observed between breech-presenting and vertex-presenting infants. Abnormal findings were present in 5.1% of infants in both presentation groups (*p* = 1000).

Likewise, no significant association between delivery mode and abnormal hip findings was found within the breech-vertex group (*p* = 0.116).

When all breech-presenting infants were compared across the different twin groups, abnormal hip findings were significantly less common in the breech-breech group than in the breech-transverse group (*p* = 0.030). No significant association with delivery mode was observed in this subgroup analysis (*p* = 0.237).

## Discussion

Breech presentation is widely recognized as a major risk factor for DDH in singleton pregnancies [3–7]. As mechanical and positional intrauterine factors are believed to contribute to abnormal hip development, twin pregnancies with reduced intrauterine space might also be expected to demonstrate increased rates of abnormal hip findings; however, in the present cohort, breech presentation in twins was not associated with an increased rate of abnormal hip ultrasound findings.

Importantly, no infant in our cohort demonstrated manifest DDH defined as Graf type IIc or higher. Only physiologically immature or mildly abnormal findings (Graf type IIa) were observed in 5.4% of infants. This distinction is important, as Graf type IIa hips may mature spontaneously during the first weeks of life and do not necessarily represent persistent DDH.

Interestingly, the breech-transverse group demonstrated significantly more abnormal ultrasound findings than the breech-breech group; however, within the breech-transverse group, no significant difference was found between breech-presenting and transverse-presenting infants. The reasons for this finding remain unclear. It is possible that the combination of noncephalic presentations reflects a particularly restricted intrauterine environment, although this assumption remains speculative and should be interpreted cautiously given the limited sample size.

Previous studies suggested that breech presentation in twins may differ mechanically from breech presentation in singleton pregnancies. Rühmann et al., referring to observations by Fettweis, described that twins in breech presentation more frequently present with flexed hips and flexed knees, whereas singleton breech presentations are more commonly associated with extended knees [20, 21]. Prolonged extension of the knees and increased tension of the ischiocrural musculature have been proposed as possible contributing factors to DDH in frank breech presentation. Furthermore, twins may turn into breech presentation later during gestation than singleton fetuses, potentially reducing the duration of mechanical stress on the hip joints [21]. These mechanisms might partially explain the comparatively low rate of abnormal findings observed in our cohort.

With respect to delivery mode, infants delivered via cesarean section demonstrated a higher rate of abnormal hip ultrasound findings than vaginally delivered infants; however, this finding should be interpreted cautiously because DDH is considered a developmental process that predominantly originates prenatally rather than during delivery itself. Therefore, delivery mode may influence postnatal hip instability or the detection of pre-existing abnormalities rather than the development of DDH. In addition, confounding factors influencing the indications for cesarean section cannot be excluded in this retrospective cohort.

Prematurity has previously been reported to influence the occurrence of DDH, particularly in singleton pregnancies [18, 22]. In our cohort, no significant differences were found between preterm and term infants, which is in line with previous observations in twin pregnancies.

Several limitations of this study must be acknowledged. First, the retrospective design limits control over potential confounding variables. Second, only infants who underwent early hip ultrasonography during the U2 examination could be included, introducing a possible selection bias. Third, no singleton control group was available. Therefore, conclusions regarding whether twin pregnancy itself influences the occurrence of DDH cannot be definitively drawn. Furthermore, information regarding family history of DDH and detailed breech subtypes was not consistently available. Follow-up examinations during the U3 screening period were also unavailable, limiting assessment of spontaneous maturation of Graf type IIa hips over time.

Despite these limitations, this study contributes additional data regarding hip ultrasound findings in twins with breech presentation, a population that remains insufficiently investigated. Further prospective studies with larger cohorts and long-term follow-up are needed to better define the relationship between twin pregnancy, fetal presentation and DDH.

## Conclusion

In this retrospective cohort of twins with at least one breech-presenting infant, breech presentation was not associated with an increased rate of abnormal hip ultrasound findings; however, the combination of breech and transverse presentation may represent a higher risk constellation. Due to the retrospective design and absence of a singleton control group, further prospective studies are required to better define the relationship between twin pregnancy, fetal presentation and developmental dysplasia of the hip.

## Data Availability

The data that support the findings of this study are available from the corresponding author upon reasonable request.

## Abbreviations

DDH: Developmental dysplasia of the hip
FUH: Frankfurt University Hospital
U2: German neonatal screening examination
U3: German infant screening examination

## Declarations

All procedures performed in studies involving human participants or on human tissue were in accordance with the ethical standards of the institutional and/or national research committee and with the 1975 Helsinki declaration and its later amendments or comparable ethical standards. Informed consent was obtained from all individual participants included in the study.
